# Lifetime Mental Health Problems in Adult Lower Secondary Education: A Student Survey

**DOI:** 10.3389/fpsyg.2020.01522

**Published:** 2020-07-06

**Authors:** Maite Aznárez-Sanado, Raquel Artuch-Garde, Sarah Carrica-Ochoa, Carlos García-Roda, Araceli Arellano, David Ramírez-Castillo, Gonzalo Arrondo

**Affiliations:** ^1^School of Education and Psychology, University of Navarra, Pamplona, Spain; ^2^Mind-Brain Group, Institute for Culture and Society, University of Navarra, Pamplona, Spain; ^3^Faculty of Education, Department of Educational Psychology and Psychobiology, Universidad Internacional de la Rioja, Logroño, Spain; ^4^National Distance Education University, Associate Center, Pamplona, Spain

**Keywords:** vocational education, adult education, mental health, psychiatric disorders, adult lower secondary education

## Abstract

**Background/Objective:**

Adult Lower Secondary Education is an education program for basic qualifications for the labor market. Our study aimed to compare lifetime mental health problems between current Adult Lower Secondary Education students and higher Vocational Education students, as the former constitutes a highly distinct and understudied group.

**Methods:**

Findings were based on a cross-sectional self-report survey. Lifetime relative odds of occurrence of mental disorders [i.e., psychiatric disorders typically diagnosed in adults, learning difficulties or deficit hyperactivity disorder (ADHD)] were compared between Adult Lower Secondary Education students (*n* = 134) and Vocational Education students (*n* = 149).

**Results:**

While the frequency of mental health problems was high in both groups, psychiatric disorders typically diagnosed in adults were more common in Adult Lower Secondary Education students than among other students. Vocational Education students reported higher rates of ADHD.

**Conclusion:**

There is a need for additional psychological resources for Adult Lower Secondary Education students, an educational level that is the last path for many to acquire a basic degree.

## Introduction

Attaining compulsory education is a key gateway for the job market and any person without such education is at a high risk of social exclusion. Adult Lower Secondary Education (ALSE) programs become the last option for many to get basic qualifications either before beginning the search for a job or even after many years of labor (Feito Alonso, 2015). We refer to ALSE as any program of instruction designed to prepare adults for the award of a lower secondary education degree (obtained in Spain after the completion of the four post-primary education courses, typically at age of 16). ALSE students constitute a highly distinct and understudied population with a history of past educational failure who nevertheless desire to continue studying. Qualitative studies on ALSE programs have shown that, although students have very varied profiles, the presence of mental disorders, disabilities and learning difficulties was a recurrent theme in their educational history (Feito Alonso, 2015). There are few studies on the frequency of mental health problems in this population. Moreover, the relationship between mental health and education is complex. Mental health issues might lead to educational difficulties and educational difficulties might lead to mental health issues. As a result, a cycle with deleterious consequences can be created for the individual.

Additionally, the age between 14 and 25 is the time when many mental health problems are first diagnosed (World Health Organization, 2013). A big portion of ALSE students are in this age range and may have specific mental health trajectories when compared to other students in the educational system. Several large studies have compared the mental health of university students against non-students of the same age (Sellström et al., 2011; Auerbach et al., 2017) and have shown that there is a lower prevalence of mental health problems in undergraduates than in individuals that are not studying, especially if they also do not work (Kovess-Masfety et al., 2016). This, nevertheless, has not been a universal finding (Alonso et al., 2004).

The risk of unemployment in Spain goes in hand with low educational levels in 2018, unemployment rates for adults with Lower Secondary Education or below was of 21%, nearly doubling the average of European countries (OECD, 2019). As for people with mental health problems, their access to jobs is not easy: only 5% are regularly employed in Spain (Muñoz et al., 2009). In this context, ALSE centers handle a big challenge in the integration of their students into the labor market (OECD, 2015). In Europe, lower secondary education is typically compulsory until the age of 16. Thus, the number of adults who have not completed this educational level is limited [less than 20% in younger adults (ages 25–34) on average across OECD countries] (OECD, 2018). Therefore, the study of prevalence of mental disorders in this group has been hampered. Indeed, samples under the age of 25 with uncompleted lower secondary education have been relatively small even in the largest surveys. For example, Auerbach et al. (2016) pooled data from over 5,000 individuals under the age of 22 that were included in the World Health Organization (WHO) mental health surveys of different countries. After combining data from different samples, there were only 157 individuals with uncompleted secondary studies in Europe. Moreover, comparisons between this group and either college students or students with completed secondary studies were not carried out. There was some indication that this was a key comparison, because the mental health of college students and secondary graduates were very similar, but there were marked differences between college students and individuals that never entered college. Similarly, Kovess-Masfety et al. (2016) included data from 2,424 French adults under the age of 24 and compared mental health of college students, secondary students (including secondary school pupils and apprentices), working individuals, and individuals who were neither employed nor students or trainees (i.e., NENST). The overall group of secondary students had a sample size of 386 individuals and the NENST group had a sample size of 266 individuals. This latter group had the worst outcomes in the study. Moreover, mental distress has been shown to be on the rise among young adults (Ross et al., 2017) and the most relevant studies use data from old surveys. WHO surveys, for example, were mostly carried out between the years 2001 and 2005 (Auerbach et al., 2016) whereas the data in Kovess-Masfety et al. (2016) was based on data from 2005. Therefore, the acquisition of new data is likely an area of great interest for researchers and practitioners.

Summarizing profiles of non-traditional learners, specifically adults, is still a scarcely studied subject (Gerber, 2012; Rothes et al., 2017). More research is needed in order to explain different trajectories of these learners, such as those enrolled in ALSE, compared to other groups. Both practice and research suggest that they may have different needs, demands, and abilities (Kaufman et al., 2008). The main objective of the present work was to assess the mental health status of ALSE students. In addition, we were also interested in assessing specific difficulties that ALSE students face in their education. For this purpose, a comparison group of higher vocational education (VE) students was also evaluated. The latter group was selected because all students would also be adults and part of a higher education itinerary (as opposed to lower levels of VE from Spain). On the other hand, less favored social classes are more likely to enter VE than more privileged classes, who choose university education more frequently (Vallejo and Dooly, 2013). Since ALSE students are potential candidates for social exclusion, higher VE students were expected to be a better comparison group than university students.

To the best of our knowledge, this is the first study comparing the occurrence of lifetime mental disorders between a sample of current ALSE students and a sample of higher VE students. Results could have organizational implications as they could help to understand some of the specific difficulties that ALSE students face in their education.

## Materials and Methods

### Procedure

The study and its consent procedure were approved by the ethics committee of the University of Navarra and carried out following the 1964 Helsinki declaration and its later amendments. All participants provided written consent. Capacity to consent was assumed for any student following the ALSE or VE curricula after a researcher duly explained the study and resolved any doubts raised in the class. As part of the safety protocol for students with mental health problems, a psychologist or educator from the research team was always present during data collection. All the participants were given the same instructions and completed the questionnaires under the same conditions. Data was acquired in November 2016 in two local schools in Pamplona (Spain), one school for each group. The ALSE school was selected as it is the only one in the capital city and the biggest of the region. Therefore, the sample is likely representative of the ALSE population, at least for larger cities. The VE school was selected for convenience reasons.

### Participants

**FIGURE 1:**
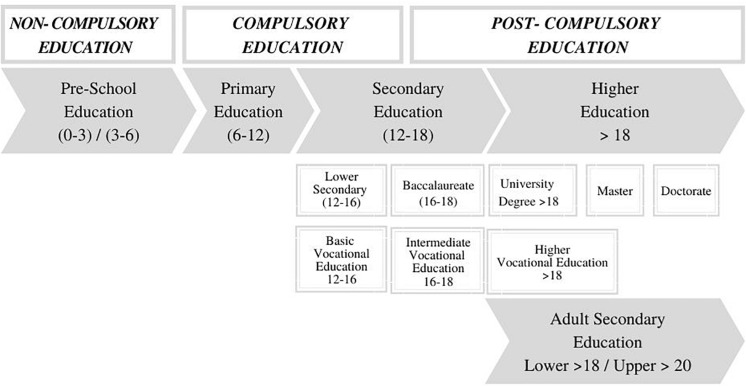
Overview of the Spanish education system.

There are 6 years of primary education in Spain, starting at age of six. After completing these academic years, students will study four courses of compulsory lower secondary education (working is not allowed until the age of 16). Most students will complete this compulsory lower secondary education and then choose either a 2-year baccalaureate or intermediate VE. The obtainment of the baccalaureate diploma permits either college entry after passing a national entry exam or, alternatively, access to higher VE. VE is one of the secondary educational options that a Spanish student is offered. There are two types of post-lower secondary VE itineraries: intermediate and higher education. To access intermediate VE a student needs to be in possession of the lower secondary education title (typically obtained at the age of 16). Higher VE can be accessed after 2 years of either baccalaureate or mid-level VE and, hence, it is typically initiated at the age of 18. It consists of two courses and it is considered a form of higher education that provides access to specialized positions in the labor market or, less frequently, to university degrees (Ministerio de Educación y Formación Profesional, 2018a; see Figure 1). In the 2016/2017 academic year, there were 313,180 students in intermediate VE and 326,982 in higher VE in Spain. ALSE in Spain is an alternative pathway to obtain the title of lower secondary education for those over the age of 18. Hence, it becomes the last option to obtain a basic educational degree for many students with a previous history of educational failure and/or who left school early. In the 2016/2017 academic year, 124,092 individuals were studying lower secondary education in this modality, accounting for 7% (Ministerio de Educación, 2017) of the overall students of lower secondary education. ALSE in Spain comprises four academic years, each covering the contents of the four courses of compulsory lower secondary education. ALSE can be offered either in-person (students attend class every day) or through distance-learning modalities (Ministerio de Educación y Formación Profesional, 2018b).

A cross-sectional self-reported survey was completed by students who were enrolled in: (1) any of the four courses of the in-person ALSE curricula or (2) higher VE (convenience control group). Students on a study break were not included. There were no other inclusion or exclusion criteria, as any student present during the days of the evaluation was offered to participate. Samples were obtained using convenience sampling. A representative sample of the day-to-day of the school was approached. After presenting the research study, four ALSE and one VE students declined to participate. A total of 134 ALSE students and of 149 VE students from two local schools in the region’s capital (out of 1 ALSE and 16 VE schools in the capital) were enrolled in the study. ALSE participants accounted for 27.6% of the total ALSE students in the region following the in-person program; the VE sample included 4.3% of higher VE students enrolled in the in-person program (Ministerio de Educación y Formación Profesional, 2019).

Higher VE was considered the best comparison group among the different levels of the educational system because they are also adults at a non-university level, and differences in demographic variables were expected between groups.

### Materials

The survey included *ad-hoc* questions on demographics and mental health. The socio-demographic questionnaire was created by the researchers for the study and included multiple-choice questions on age (coded as between 18 and 25, 26–30, 31–35, 36–40, or over 40), sex, cultural origin, family, level of parental education, educational history, work status, and mental health.

Regarding mental health, participants were asked to indicate if they had ever been diagnosed with disorders by a health professional. In this study, mental health problems were divided into three main groups: (1) Mental disorders typically diagnosed during adolescence or adulthood (i.e., depression, anxiety, social phobia, OCD, personality disorder, eating disorder, schizophrenia, bipolar disorder, PTSD, substance abuse, and problem gambling). These categories were the same as the ones included in the MINI International Neuropsychiatric Interview (Lecrubier et al., 1998) and they corresponded to the most frequent mental disorders; (2) Four problems that mainly or heavily affect school results (i.e., dyslexia, dyscalculia, language disorder, and intellectual disability) were also investigated; (3) Finally, ADHD was included as a stand-alone disorder. Whereas it is typically diagnosed in childhood, ADHD symptoms continue to adulthood and, in many cases, its consequences reach far over than school results. Moreover, ADHD is highly comorbid with many disorders included in the first or second groups.

We chose lifetime diagnoses because mental health problems in childhood and adolescence are predictors of work incapacity across the lifespan (Goodman et al., 2011). Additionally, lifetime existence of mental health problems is better suited to compare small groups or low frequency disorders as overall numbers will be greater.

The study also included the Spanish versions of the Motivated Strategies for Learning Questionnaire (Pintrich, 1991; Roces et al., 1995), the Discomfort Intolerance Scale (Harrington, 2005; Medrano et al., 2018) and a modified version of a questionnaire on perception of competency for employment (Martínez-Rodríguez and Carmona, 2010). Results from these questionnaires, along those of many of the questions of the sociodemographic questionnaire were aimed at providing additional insights of the samples and will not be reported here. All instruments were answered by participants in optical automatic reading response sheets.

### Analyses

The percentage of female individuals, of participants under the age of twenty-five, those from a foreign country, and that had a parent with a post-secondary degree or superior educational level were obtained for each of the educational groups and compared between ALSE and VE using Chi-squared tests. The number of subjects who had ever received a diagnosis of any of the mental disorders were identified in each of the groups (ALSE, VE). Observations were independent because each participant was only counted as one observation. Variables in our study were dichotomous and presented a binomial distribution. Odds of a previous diagnosis of a mental disorder were then compared between ALSE and VE (reference group) students. Odds ratios (OR) and 95% confidence intervals were calculated based on exact logistic regression analyses [dependent variable = lifetime history of mental disorder, independent variable = group (ALSE, VE)] reporting a measure of association between mental health and educational level. ORs were calculated for: (1) any and each of 11 mental disorders typically diagnosed in adulthood, (2) ADHD, and (3) any and each of the other learning difficulties.

ORs including all ALSE and VE students were calculated. As expected, socio-demographic differences were found between groups. Almost all individuals (95.3%) in VE were under the age of 25, and this was not the case in ALSE (∼40% of students were over 25 years old). Individuals under the age of 25 also differed in the proportion of men and women in ALSE and VE (42.7% female in ALSE and 28.9% female in VE). Because sex and age are important risk factors for many mental health problems, we were interested in exploring the effect of these confounding variables in our results. For this purpose, ORs including only individuals under the age of 25 were calculated in another analysis. Additionally, we obtained ORs in those subjects who were under the age of 25, including sex as a covariate in the logistic regression analysis.

Statistical analyses were performed in Stata v13 and *p*-values under 0.05 were considered significant.

## Results

The ALSE and VE groups differed significantly in key socio-demographic variables. More students in ALSE: (1) were over the age of 25 [38.8 vs. 4.7% in VE, χ^2^(1) = 44.06, *p*< 0.001], (2) were female [49.3 vs. 30.9% in VE, χ^2^(1) = 9.97, *p*< 0.01], (3) were born in a foreign country [51.9 vs. 14.1%, χ^2^(1) = 46.17, *p*< 0.001], and (4) their parents had lower education grades [57% of students in ALSE had a parent with a post-secondary degree vs. 81.9% in VE, χ^2^(1) = 20.40, *p*< 0.001]. Additionally, socio-demographic variables related to subjects with a lifetime history of mental disorders can be found in Tables 1, 2.

**TABLE 1:** Lifetime mental health problems in Adult Lower Secondary Education and Vocational Education.

	ALSE (*n* = 134)						VE (*n* = 149)						
	Cases (*n*)	%	>25 (%)	FM (%)	FR (%)	P.E. (%)	Cases (*n*)	%	>25 (%)	FM (%)	FR (%)	P.E. (%)	Raw OR (95% CI)
Any mental health problem of adulthood^b^	36	26.7	44.4	69.4	41.7	77.8	19	12.8	5.3	26.3	26.3	89.5	2.5 (1.31–4.92)*
Depression	21	15.7	47.6	76.2	52.4	76.2	7	4.7	0.0	57.1	28.6	85.7	3.75 (1.47–10.83)*
Anxiety	23	17.2	34.8	60.9	26.1	78.3	8	5.4	0.0	37.5	25.0	87.5	3.63 (1.50–9.78)*
Social phobia	5	3.7	20.0	80.0	0.0	60.0	0	0.0	–	–	–	–	7.68 (1.03–Inf)*
OCD	5	3.7	40.0	40.0	0.0	100.0	2	1.3	0.0	0.0	50.0	100.0	2.83 (0.45–30.29)
Personality disorder	6	4.5	16.7	66.7	50.0	83.3	2	1.3	0.0	0.0	50.0	100.0	3.43 (0.60–35.34)
Eating disorder	5	3.7	40.0	100.0	20.0	80.0	4	2.7	25.0	75.0	50.0	75.0	1.40 (0.29–7.22)
Schizophrenia	1	0.8	0.0	0.0	100.0	100.0	1	0.7	0.0	0.0	0.0	100.0	1.11 (0.01–87.93)
Bipolar disorder	1	0.8	0.0	100.0	0.0	100.0	0	0.0	–	–	–	–	1 (0.95–Inf)
PTSD	3	2.2	33.3	66.7	66.7	66.7	1	0.7	0.0	0.0	0.0	100.0	3.37 (0.27–179.11)
Substance abuse	3	2.2	33.3	66.7	66.7	66.7	4	2.7	0.0	0.0	50.0	100.0	0.83 (0.12–5.01)
Problem gambling	0	0.0	–	–	–	–	3	2.0	0.0	0.0	33.3	66.7	0.29 (0.00–2.68)
ADHD	11	8.2	27.3	45.5	36.4	54.5	25	16.8	4.0	4.0	12.0	100.0	0.44 (0.19–0.99)*
Other learning difficulties^c^	11	8.2	45.5	45.5	54.5	72.7	9	6.0	0.0	33.3	11.1	100.0	1.39 (0.50–3.93)
Dyslexia	2	1.5	50.0	50.0	100.0	100.0	3	2.0	0.0	66.7	33.3	100.0	0.74 (0.06–6.54)
Dyscalculia	5	3.7	40.0	60.0	60.0	60.0	2	1.3	0.0	50.0	50.0	100.0	2.84 (0.45–30.29)
Language disorder	0	0.0	–	–	–	–	4	2.7	0.0	0.0	25.0	100.0	0.21 (0.00–1.67)
Intellectual disability	4	3.0	50.0	25.0	25.0	75.0	2	1.3	0.0	0.0	0.0	100.0	2.25 (0.32–25.31)

ALSE, Adult Lower Secondary Education; VE, Vocational Education; OR, odds ratio; PTSD, post-traumatic stress disorder; OCD, obsessive-compulsive disorder; ADHD, attention deficit hyperactivity disorder. Total number of cases and percentages are reported. ORs and their respective 95% confidence intervals are calculated as a measure of association between mental health and educational level [ALSE, VE (reference group)]. Columns “>25 (%)” (percentage of subjects over 25 years old), “FM (%)” (percentage of females), “FR (%)” (percentage of students who were born in a foreign country), and “P.E. (%)” (percentage of students who had at least a parent with post-secondary degree or superior educational level) represent sociodemographic data of subjects who reported a lifetime history of mental disorders. *Indicating significant results (p < 0.05). ^*b*^Any of the 11 common mental disorders typically diagnosed in adulthood: depression, anxiety, social phobia, OCD, personality disorder, eating disorder, schizophrenia, bipolar disorder, PTSD, substance abuse, and problem gambling. ^*c*^Any of the 4 learning difficulties: dyslexia, dyscalculia, language disorder, and intellectual disability.

**TABLE 2:** Lifetime mental health problems in Adult Lower Secondary Education and Vocational Education, including only subjects under 25.

	ALSE ≤ 25 (*n* = 82)					VE ≤ 25 (*n* = 142)						
	Cases (*n*)	%	FM (%)	FR (%)	P.E. (%)	Cases (*n*)	%	FM (%)	FR (%)	P.E. (%)	Raw OR ≤ 25	Adjusted OR ≤ 25^a^
Any mental health problem of adulthood^b^	20	24.4	75.0	45.0	95.0	18	12.7	22.2	22.2	88.9	2.21 (1.03–4.80)*	2.01 (0.92–4.40)
Depression	11	13.4	81.8	45.5	90.9	7	4.9	57.1	28.6	85.7	2.97 (1.01–9.46)*	2.44 (0.79–8.00)
Anxiety	15	18.3	66.7	33.3	93.3	8	5.6	37.5	25.0	87.5	3.72 (1.40–10.69)*	3.32 (1.23–9.63)*
Social phobia	4	4.9	75.0	0.0	75.0	0	0.0	–	–	–	9.46 (1.17–Inf)*	7.68 (0.93–Inf)
OCD	3	3.7	66.7	0.0	100.0	2	1.4	0.0	50.0	100.0	2.65 (0.30–32.30)	2.59 (0.28–32.13)
Personality disorder	5	6.1	60.0	60.0	100.0	2	1.4	0.0	50.0	100.0	4.51 (0.72–48.45)	4.37 (0.68–47.50)
Eating disorder	3	3.7	100.0	0.0	100.0	3	2.1	66.7	33.3	66.7	1.75 (0.23–13.42)	1.31 (0.16–10.33)
Schizophrenia	1	1.2	0.0	100.0	100.0	1	0.7	0.0	0.0	100.0	1.74 (0.02–137.6)	2.16 80.27–172.31)
Bipolar disorder	1	1.2	100.0	0.0	100.0	0	0.0	–	–	–	1.73 (0.04–Inf)	1.17 (0.03–Inf)
PTSD	2	2.4	50.0	50.0	100.0	1	0.7	0.0	0.0	100.0	3.5 (0.18–209.40)	3.59 (0.18–218.70)
Substance abuse	2	2.4	50.0	100.0	100.0	4	2.8	0.0	50.0	100.0	0.86 (0.08–6.17)	0.96 (0.08–7.00)
Problem gambling	0	0.0	–	–	–	3	2.1	0.0	33.3	66.7	0.45 (0.00–4.19)	0.55 (0.00–5.21)
ADHD	8	9.8	50.0	25.0	62.5	24	16.9	4.2	12.5	100.0	0.53 (0.20–1.31)	0.60 (0.22–1.49)
Other learning difficulties^c^	6	7.3	33.3	66.7	83.3	9	6.3	33.3	11.1	100.0	1.17 (0.33–3.83)	1.17 (0.33–3.90)
Dyslexia	1	1.2	0.0	100.0	100.0	3	2.1	66.7	33.3	100.0	0.57 (0.01–7.27)	0.51 (0.01–6.68)
Dyscalculia	3	3.7	66.7	66.7	66.7	2	1.4	50.0	50.0	100.0	2.64 (0.30–32.30)	2.29 (0.25–28.52)
Language disorder	0	0.0	–	–	–	4	2.8	0.0	25.0	100.0	0.32 (0.00–2.61)	0.40 (0.00–3.24)
Intellectual disability	2	2.4	0.0	50.0	100.0	2	1.4	0.0	0.0	100.0	1.74 (0.12–24.5)	2.18 (0.15–31.06)

ALSE, Adult Lower Secondary Education; VE, Vocational Education; ALSE ≤ 25 and VE ≤ 25, individuals under the age of 25 in ALSE and VE, respectively; OR, odds ratio; PTSD, post-traumatic stress disorder; OCD, obsessive-compulsive disorder; ADHD, attention deficit hyperactivity disorder. Total number of cases and percentages are reported. ORs and their respective 95% confidence intervals are calculated as a measure of association between mental health and educational level [ALSE, VE (reference group)]. Columns “FM (%)” (percentage of females), “FR (%)” (percentage of students who were born in a foreign country), and “P.E. (%)” (percentage of students who had at least a parent with post-secondary degree or superior educational level) represent sociodemographic data of subjects who reported a lifetime history of mental disorders. *Indicating significant results (p < 0.05). ^*a*^Controlled for sex. ^*b*^Any of the 11 common mental disorders typically diagnosed in adulthood: depression, anxiety, social phobia, OCD, personality disorder, eating disorder, schizophrenia, bipolar disorder, PTSD, substance abuse, and problem gambling. ^*c*^Any of the 4 learning difficulties: dyslexia, dyscalculia, language disorder, intellectual disability.

Lifetime, self-reporting of mental health problems was high. For example, 27% of individuals in ALSE reported a history of mental health problems of adulthood, and 17% of VE students reported a history of ADHD. Students in ALSE showed a significantly higher probability of having a history of mental health problems typically diagnosed in adulthood, which was mainly attributable to an increased probability of being diagnosed with depression and anxiety (Table 1). Students in VE reported higher rates of previous diagnoses of ADHD, although differences were not statistically significant when controlling for age and sex.

Lastly, comorbidity was found among participants. We found that the number of students who ever received a diagnosis of at least one of the mental disorders was 48 in ALSE and 46 in VE. Out of these, 21 subjects in ALSE and 10 subjects in VE showed a positive diagnosis for more than one mental disorder.

## Discussion

Our study showed that present or past mental health issues in students enrolled in ALSE are frequent and, indeed, more prevalent than in other forms of under-college adult education. Lifetime risk of depression and anxiety in VE was close to that found in college students worldwide as seen in the World Health Organizations surveys. However, these same rates were much higher in ALSE students than in students that did not enter college in the WHO surveys (Auerbach et al., 2017) even when only students under age of 25 were considered. This reaffirms the distinct status of ALSE enrollees compared to other students of the education system. Whereas several comparisons did not reach statistical significance when controlling both by age and sex, ORs still suggested differences between the groups. Hence, the lack of statistical significance likely results from the reduced power of the comparison to detect effects.

Unexpectedly, frequency of ADHD reporting was more common in VE students than in ALSE students, although this difference was not statistically significant when controlling for age. Lifetime self-reported diagnosis of ADHD has been estimated to occur in 11% of individuals under 18 (National Institute of Mental Health, 2017) a rate close to the one found in ALSE students, but much lower than that of VE students. Both the high frequency of reporting and the counter-intuitive difference between groups open future paths of research. It could be hypothesized that such patterns might be related to generational issues on the diagnosis of ADHD. Alternatively, some aspects related to the demographics of ALSE students, such as an origin in foreign countries with different health systems, might also be influencing the rates of ADHD diagnoses.

The fact that we used lifetime diagnoses should also be stressed. We cannot know if individuals who received a diagnosis in the past had a current episode. Nevertheless, having a mental health disorder at any point of the educational life course could potentially have some relevant impact years after. Additionally, the fact that diagnoses were self-reported could lead to recall bias. However, other noteworthy studies such as the National Health Interview Survey (NHIS) in the US have used, similarly, posed questions for over 50 years. In our case, such a design permitted the concurrent evaluation of whole classes in a short period of time. There are several possible explanations for the greater risk of mental health diagnoses in ALSE students. First, the results could be simply attributable to age differences between groups. ALSE students have are older than other type of students and, therefore, have a greater cumulative time to receive a diagnosis. However, the fact that most differences remained after controlling for age is evidence against this hypothesis. Similarly, other demographics could also be related to greater mental health diagnoses in the ALSE group. For example, living in a foreign country or having a lower socioeconomic status are both related to higher rates of psychiatric disorders. ALSE and VE students differ on many sociodemographic variables, any of which could be driving the differences in reported psychopathology. However, the fact that the origin of these differences cannot be known from our analyses should not distract from the importance of our results. From an organizational perspective, higher frequencies of self-reported psychopathology, independent of their cause, could indicate a necessity for more educational or psychological resources in these programs. Second, past or chronic mental health problems are likely bidirectionally linked with educational difficulties: mental health problems could lead to educational difficulties and likewise, educational difficulties could lead to mental health problems. Therefore, mental health issues would be related to higher rates of dropouts in the educational system and, indirectly, to greater rates when individuals come back to ALSE programs. Finally, ALSE programs might be seen as an interesting resource for a minority of individuals with current mental health problems. Some individuals may have difficulties finding a job due to their specific handicaps, and ALSE programs might be used as some form of occupational therapy.

Our study has several important limitations. First, mental health assessment through self-report scales is not ideal and there are other preferred methods, such as clinical interviews. However, such instruments also increase administration time. Participants’ responses might be biased due to the presence of a person from the research team during data collection. In addition, it is important to note that ORs corrected by age are not representative of the whole ALSE population, because they only include young adults. Sample sizes might not have been large enough to reliably study differences between ALSE and VE groups in the less common diagnoses. According to power analyses, our data lacked statistical power to detect differences between groups in mental health problems which were smaller than 5%. To minimize this issue, we used both lifetime frequency of diagnoses and groups of disorders to increase counts in each of the groups. In this case we traded specificity for improved reliability. Moreover, samples are probably not fully representative of their respective populations because they were recruited from only one institution. This is likely more marked in the case of VE than ALSE, as in ALSE groups, we evaluated a large proportion of the students in a key school in the region. It could also be argued that ALSE students form a pre-selected sample. Those who enrolled ALSE are those who, for some reason, decided that it was important for them to obtain a lower secondary education degree. However, some percentage of students who dropped out do not continue with their education, and they were not part of our research. Because our results cannot be generalized to all students that dropped out from the educational system, it is highly relevant to describe the ALSE population as they likely differ both from other students (as shown here) and other individuals who never come back to the educational system. Future studies could aim to compare ALSE to this latter group. Finally, it is important to note that distance learning students were not included in the present work. Future research should take this population into consideration.

Despite the limitations derived from being a small survey, our study highlights the need for additional educational, psychological, and economic resources in an understudied educational level that is the last path for many to acquire a basic degree. There are higher rates of mental health problems in ALSE and this will influence day-to-day school interactions. According to previous work, teachers usually play a key role in the detection of mental health problems and in students’ well-being (Wang et al., 2013; Brandseth et al., 2019). However, research also indicates that teaching is a highly demanding career (Yang et al., 2019) with teachers reporting high rates of poor mental health (McLean et al., 2017). Further research should investigate the relationship between psychosocial factors at schools and mental well-being of teachers and students. In order to respond effectively to students’ needs, teachers’ psychological support and training are vital. Internal and external guidance departments should play a key role in this labor.

Whole-school mental health promotion programs have demonstrated some efficacy (Kern et al., 2017; O’Reilly et al., 2018). They are probably more cost-effective in settings with high prevalence of mental health issues, and specially for the prevention of anxiety and depression (Mendelson and Eaton, 2018). These two disorders were found to be most frequent in ALSE compared to VE. ALSE might be an ideal setting to benefit from these programs. An interpretation of the higher prevalence of mental health problems among students attending ALSE could be that students who have mental health issues drop out from the mainstream education and eventually must enroll in ALSE programs. Therefore, mental health hygiene in regular education could also lead to fewer dropouts and, indirectly, fewer students taking the ALSE route because of mental health problems. Larger epidemiological studies on mental health of ALSE students in developed countries are needed for an improved understanding of the needs of ALSE students.

## Data Availability Statement

Participant-level data and further information on data acquisition procedure have been uploaded to an online repository (Aznárez-Sanado et al., 2020).

## Ethics Statement

The studies involving human participants were reviewed and approved by the Ethics committee of the University of Navarra. The patients/participants provided their written informed consent to participate in this study.

## Author Contributions

MA-S and GA analyzed the data and drafted the initial version of the article. All authors designed the study, obtained funding, acquired the data, and contributed to its interpretation and approved the final version.

## Conflict of Interest

The authors declare that the research was conducted in the absence of any commercial or financial relationships that could be construed as a potential conflict of interest.
